# Determination of clinical and demographic predictors of laboratory-confirmed influenza with subtype analysis

**DOI:** 10.1186/1471-2334-12-129

**Published:** 2012-06-07

**Authors:** Tabitha Woolpert, Stephanie Brodine, Hector Lemus, Jill Waalen, Patrick Blair, Dennis Faix

**Affiliations:** 1 Naval Health Research Center, 140 Sylvester Rd, San Diego, CA, 92106, USA; 2 San Diego State University, Hardy Tower 119, 5500 Campanile Dr, San Diego, CA, 92182-4162, USA; 3 UCSD School of Medicine, 9500 Gilman Drive #0811, La Jolla, CA, 92093-0811, USA

**Keywords:** Influenza, Influenzavirus A, Influenzavirus B, Influenza A Virus, H1N1 Subtype, Diagnosis

## Abstract

**Background:**

Rapid influenza diagnosis is important for early identification of outbreaks, effective management of high-risk contacts, appropriate antiviral use, decreased inappropriate antibiotic use and avoidance of unnecessary laboratory testing. Given the inconsistent performance of many rapid influenza tests, clinical diagnosis remains integral for optimizing influenza management. However, reliable clinical diagnostic methods are not well-established. This study assesses predictors of influenza, and its various subtypes, in a broad population at the point of care, across age groups, then evaluates the performance of clinical case definitions composed of identified predictors.

**Methods:**

Respiratory specimens and demographic and clinical data were obtained from 3- to 80-year-old US military family members presenting for care with influenza-like illness (ILI) from November 2007 to April 2008. Molecular and virus isolation techniques were used to detect and subtype influenza viruses. Associations between influenza diagnosis and demographic/clinical parameters were assessed by logistic regression, including influenza type and subtype analyses. The predictive values of multiple combinations of identified clinical predictors (case definitions), and the Centers for Disease Control and Prevention (CDC) ILI case definition, were estimated.

**Results:**

Of 789 subjects, 220 (28%) had laboratory-confirmed influenza (51 A(H1), 46 A(H3), 19 A(unsubtypeable), 67 B, 1 AB coinfection), with the proportion of influenza A to B cases highest among 6- to 17-year-olds (*p* = 0.019). Independent predictors of influenza included fever, cough, acute onset, body aches, and vaccination status among 6- to 49-year-olds, only vaccination among 3- to 5-year-olds, and only fever among 50- to 80-year-olds. Among 6- to 49-year-olds, some clinical case definitions were highly sensitive (100.0%) or specific (98.6%), but none had both parameters over 60%, though many performed better than the CDC ILI case definition (sensitivity 37.7%, 95% confidence interval 33.6–41.9% in total study population).

**Conclusions:**

Patterns of influenza predictors differed across age groups, with most predictors identified among 6- to 49-year-olds. No combination of clinical and demographic predictors served as a reliable diagnostic case definition in the population and influenza season studied. A standardized clinical case definition combined with a point-of-care laboratory test may be the optimal rapid diagnostic strategy available.

## Background

Influenza infections result in 3–5 million illnesses and 500,000 deaths each year worldwide [1]. The US records an average of 226,000 hospitalizations and 36,000 deaths annually [2]. Because of high mutability, cross-species transfer, and reservoir diversity, influenza is not considered eradicable [3-5]; effective prevention and treatment are needed to mitigate its impact. Prompt and accurate diagnosis facilitates early identification of outbreaks, timely intervention, effective management of high-risk contacts, appropriate antimicrobial use, and avoidance of unnecessary laboratory testing [6]. Recent experience with pandemic H1N1 influenza (pH1N1) [7,8] highlights the need to reevaluate current diagnostic capabilities and seasonal influenza’s clinical presentation.

Polymerase chain reaction (PCR) assays discriminate between influenza types and subtypes and are more sensitive (83%–96%) than culture (44%–57%) [9-11]; PCR typically requires up to 24 hours for results; culture can require 10 days. Rapid influenza detection tests (RIDTs) are available with high specificities (96%–100%) but poor sensitivities (27%–69%) [12-15]. Lacking rapid assays that are consistently sensitive, a reliable model for predicting influenza infection based on clinical and demographic parameters would be valuable.

Multiple studies have evaluated clinical case definitions (clinical and demographic parameter combinations) as diagnostic tools [16-30]. Interpretation and application of these findings is hampered by differing methodologies, disparate clinical settings, varying inclusion criteria, a wide range of influenza prevalence, multiple outcome measures, and virus evolution. Approximately half of these studies found clinical models to predict influenza [17,18,21,23,26,27,29-31], suggesting the presence of fever, cough, and acute onset might accurately identify influenza during a local epidemic. Few prior studies analyzed the effect of age on clinical predictors, although two studies suggested predictor models are age dependent [27,30]. A 2005 meta-analysis highlighted the non-standardization problem when it found only ten of 915 studies on symptom-based influenza diagnosis from 1966 to 2004 to meet the following criteria: (1) prospective design or randomized trial, (2) inclusion of primary assessment of clinical predictors of influenza, (3) use of standard laboratory-based outcome, and (4) high study quality based on a previously published scheme [18]. A 2011 systematic review covering a similar time frame identified 12 studies meeting similar criteria; authors found fever plus cough and fever/cough/acute onset heuristics to have areas under the receiver operating characteristic curves of 0.70 and 0.79, respectively. Authors additionally observe that none of the more robust studies have conducted a split-sample validation nor prospectively validated a clinical decision rule [31].

We present findings that (1) identify influenza predictors in different age groups, (2) assess the diagnostic value of clinical case definitions based on these predictors, and (3) compare clinical presentation across influenza type and subtype in a local population during one influenza season.

## Methods

### Subjects

From November 2007 to May 2008 study personnel recruited eligible clinic patients with subjective fever and/or temperature >38.0 °C and either a cough or sore throat into an ongoing laboratory-based respiratory disease surveillance study. This surveillance study was established to track rates and etiologies of respiratory disease in the local military dependent population, as well as to provide an infrastructure for the evaluation of novel influenza diagnostics. Recruitment occurred in two community-based outpatient clinics and the emergency department and pediatric clinic at Naval Medical Center San Diego, all part of the regional military treatment area serving half a million Department of Defense (DoD) clients. Eligible subjects were DoD dependents or retirees ages 3–80 years who provided, or whose legal guardians provided, informed consent. Though non-participation rates could not be tracked precisely, and demographics of non-participants are not known, it is estimated that greater than 90% of those solicited were enrolled.

### Data collected

Associates interviewed each subject/guardian to determine age, sex, presence of subjective fevers, number of days since symptom onset, smoking history (pack-years), vaccine history, and presence of eight symptoms during the current illness (see Table 1). Subject temperature (oral) was obtained from the medical record.

**Table 1:** Descriptive statistics for laboratory-confirmed influenza outcomes and demographic and clinical variables

		**Subjects ages**	**Subjects ages**	**Subjects ages**	**Subjects ages**
	**Total study population**	**3–5 years**	**6–17 years**	**18–49 years**	**50–80 years**
**Variable**	**(*n* = 789)**	**(*n* = 65)**	**(*n* = 197)**	**(*n* = 326)**	**(*n* = 201)**
Outcome variables	*n* (%)	*n* (%)	*n* (%)	*n* (%)	*n* (%)
Influenza diagnosis					
Positive	220 (27.9)	12 (18.5)	65 (33.0)	92 (28.2)	51 (25.4)
Negative	569 (72.1)	53 (81.5)	132 (67.0)	234 (71.8)	150 (74.6)
Influenza type					
A	152 (69.1)	7 (58.3)	53 (81.5)	63 (68.5)	29 (56.9)
B	67 (30.5)	5 (41.7)	11 (16.9)	29 (31.5)	22 (43.1)
AB	1 (0.5)	0 (0.0)	1 (1.5)	0 (0.0)	0 (0.0)
Influenza A subtype^a^					
H1	51 (44.0)	3 (42.9)	19 (44.2)	18 (38.3)	11 (57.9)
H3	46 (39.7)	1 (14.3)	21 (48.8)	17 (36.2)	7 (36.8)
Unsubtypeable	19 (16.4)	3 (42.9)	3 (7.0)	12 (25.5)	1 (5.3)
Continuous variables	Value (error)^b^	Value (error)^b^	Value (error)^b^	Value (error)^b^	Value (error)^b^
Age (years)	31.2 (19.4)	4.0 (0.8)	11.5 (3.6)	32.0 (9.9)	56.8 (5.3)
Symptom duration (days)	4.0 (1–90)	4.0 (1–30)	3.0 (1–60)	5.0 (1–60)	5.0 (1–90)
Temperature (°C)	36.9 (35.6–40.3)	36.8 (35.9–39.5)	37.1 (35.6–40.3)	36.9 (35.9–39.6)	36.8 (35.6–39.3)
Smoking history (pack-years)	0.0 (0–117.5)^c^	––^d^	––^d^	0.0 (0–51)^c^	0.0 (0–117.5)
Vaccine to Onset Time (Days)^e^	80 (0–365)	57 (0–198)	74 (6–348)	89 (3–342)	85.4 (8–365)
Categorical variables	*n* (%)	*n* (%)	*n* (%)	*n* (%)	*n* (%)
Enrollment site					
Outpatient clinic 1	284 (36.0)	6 (9.2)	61 (31.0)	137 (42.0)	80 (39.8)
Outpatient clinic 2	203 (25.7)	15 (23.1)	55 (27.9)	63 (19.3)	70 (34.8)
Emergency room	246 (31.2)	24 (36.9)	47 (23.9)	124 (38.0)	51 (25.4)
Pediatric clinic	56 (7.1)	20 (30.8)	34 (17.3)	2 (0.6)	0 (0.0)
Sex					
Female	494 (62.6)	30 (46.2)	87 (44.2)	270 (82.8)	107 (53.2)
Male	295 (37.4)	35 (53.8)	110 (55.8)	56 (17.2)	94 (46.8)
Acute onset					
≤3 days	273 (34.6)	30 (46.2)	99 (50.3)	100 (30.7)	44 (21.9)
>3 days	516 (65.4)	35 (53.8)	98 (49.8)	226 (69.3)	157 (78.1)
Fever					
>38.0°C	126 (16.0)	11 (16.9)	48 (24.4)	43 (13.2)	24 (11.9)
≤38.0°C	663 (84.0)	54 (83.1)	149 (75.6)	283 (86.8)	177 (88.1)
Cough					
Yes	736 (93.3)	63 (96.9)	176 (89.3)	299 (91.7)	198 (98.5)
No	53 (6.7)	2 (3.1)	21 (10.7)	27 (8.3)	3 (1.5)
Sore throat					
Yes	630 (79.8)	33 (50.8)	170 (86.3)	278 (85.3)	149 (74.1)
No	159 (20.2)	32 (49.2)	27 (13.7)	48 (14.7)	52 (25.9)
Shortness of breath^e^					
Yes	346 (43.9)	20 (30.8)	73 (37.2)	166 (50.9)	87 (43.3)
No	442 (56.0)	45 (69.2)	123 (62.8)	160 (49.1)	114 (56.7)
Nasal congestion					
Yes	650 (82.4)	51 (78.5)	155 (78.7)	271 (83.1)	173 (86.1)
No	139 (17.6)	14 (21.5)	42 (21.3)	55 (16.9)	28 (13.9)
Headache					
Yes	619 (78.5)	31 (47.7)	134 (68.0)	286 (87.7)	168 (83.6)
No	170 (21.5)	34 (52.3)	63 (32.8)	40 (12.3)	33 (16.4)
Conjunctivitis^e^					
Yes	44 (5.6)	6 (9.2)	4 (2.0)	16 (4.9)	18 (9.0)
No	744 (94.3)	59 (90.8)	193 (98.0)	309 (94.8)	183 (91.0)
Body aches^e^					
Yes	581 (73.6)	27 (41.5)	99 (50.2)	289 (88.7)	166 (82.6)
No	207 (26.2)	37 (56.9)	98 (49.8)	37 (11.4)	35 (17.4)
Nausea/vomiting					
Yes	347 (44.0)	19 (29.2)	107 (54.3)	155 (47.5)	66 (32.8)
No	442 (56.0)	46 (70.8)	90 (45.7)	171 (52.5)	135 (67.2)
Smoker					
Yes	157 (19.9)	0 (0.0)	0 (0.0)	88 (27.0)	69 (34.3)
No	632 (80.1)	65 (100.0)	197 (100. 0)	238 (73.0)	132 (65.7)
Vaccinated					
Yes	217 (27.5)	17 (26.2)	27 (13.7)	85 (26.1)	88 (43.8)
No	572 (72.5)	48 (73.9)	170 (86.3)	241 (73.9)	113 (56.2)

NOTE. Percentages are rounded, thus may not add up to 100%. ^a^Denominator is subjects with at least one specimen positive by culture for influenza A. ^b^Continuous variable values reported as “mean (standard deviation)” if symmetric variable, or “median (low end–high end of range)” if nonsymmetric. ^c^Two subjects in age group reported history of smoking but datum for number of pack-years is missing. ^d^In this age group no subjects reported pack-years >0. ^e^Median (range) reported for subset of subjects who reported any influenza vaccine within the 12 months prior to presentation. ^f^Datum missing for 1 subject in study population.

### Specimen processing

Specimen collection and processing proceeded according to three schemes varying by number and type of specimens tested and whether they were frozen or processed fresh. This variation resulted from concomitant evaluation of two novel influenza diagnostics occurring in the same population, the evaluations requiring different specimen combinations. Up to two mucosal respiratory specimens (throat and/or nasal swabs) were collected for testing using Dacron swabs (Remel, Inc., MicroTest Viral Transport Media [VTM] Female Kit, Reference #12550) immediately placed into the VTM. When both a nasal and throat specimen were requested, specimens were maintained at 2–8 °C or on ice and all testing was initiated within 72 hours of collection. All other specimens were refrigerated for ≤10 hours after collection then stored at ≤ −70 °C until testing.

Each specimen was tested for influenza A by end-point reverse transcriptase PCR (RT-PCR) as previously described using primer sets 2 and 2.5 [32]. Each was also inoculated on rhesus monkey kidney cells for virus isolation [33]; cultures exhibiting cytopathic effect within 14 days were tested using an indirect immunofluorescence assay with type-specific monoclonal antibodies for viral identification. Isolates were subtyped by hemagglutination inhibition (HAI) using the World Health Organization Influenza Reagent Kit for Identification of Influenza Isolates 2007–2008 (WHO, Geneva, Switzerland). A portion of unsubtypeable specimens were tested with the T5000 PCR/electrospray ionization mass spectrometer (ESI-MS) (Ibis Pharmaceuticals, Carlsbad, CA) as previously described [34] to identify influenza subtype.

### Statistical analysis

Laboratory-confirmed influenza was defined as having any specimen positive for influenza by RT-PCR or virus isolation from cell culture. Influenza A status was determined by RT-PCR and virus isolation and influenza B status by virus isolation. An influenza A(H1) or A(H3) case was a subject with any specimen positive for that influenza subtype by HAI.

The study population was grouped into age categories: 3–5 years, 6–17 years, 18–49 years, and 50–80 years, chosen based on standard clinical groupings according to probable influenza exposure/risk environments. Age was treated as a continuous variable within each group. Dichotomous variables were constructed from all clinical parameters. Acute onset was defined as symptom onset ≤3 days before presentation. Fever was defined as temperature >38.0 °C. Since inclusion criteria required cough or sore throat, these two variables were not considered independent of each other; sore throat was excluded from the multivariate model. History of adequate vaccination was defined as receipt >14 and ≤365 days prior to symptom onset.

Rates of influenza across age groups were compared using Pearson’s chi-square test. Bivariate exact conditional regression was performed in each age group to model the probability of laboratory-confirmed influenza against age, sex, acute onset, fever, cough, sore throat, shortness of breath, nasal congestion, headache, conjunctivitis, body aches, nausea/vomiting, smoking history, and vaccination status. Age groups demonstrating similar predictor patterns were combined and bivariate analyses repeated. Backward stepwise logistic regression using all above variables except sore throat was employed to create a reduced multivariate model. Age and sex remained in the reduced model regardless of any association. The associations between influenza and both enrollment site and number/type of specimens were assessed in bivariate and multivariate models in the whole population. Both were then removed from the model because they did not alter predictor variable associations. The same clinical and demographic variables used in the primary multivariate model were evaluated for associations with laboratory-confirmed influenza A versus B and influenza A(H1) versus A(H3) in bivariate and multivariate logistic regression models in the combined age group.

Variables demonstrating associations with laboratory-confirmed influenza (ie, the predictors of influenza) in the combined age group reduced model were incorporated into various clinical case definitions classified by at least *n* criteria or by particular combinations of clinical criteria. Modeling these case definitions as diagnostic tests, four test performance parameters—sensitivity, specificity, and positive and negative predictive values (PPV, NPV)—were calculated. Performance of the Centers for Disease Control and Prevention (CDC) influenza-like illness (ILI) case definition (temperature ≥37.8 °C and either cough or sore throat) [35] was assessed for comparison. Confidence intervals (CIs) for performance parameters were calculated for binomial proportions.

All statistical analyses utilized SAS software, version 9.1 (SAS Institute, Inc., Cary, NC); *p* values < .05 were considered significant. This study was approved by the Naval Health Research Center Institutional Review Board and followed the human subject research guidelines of that institution.

## Results

Between November 14, 2007 and April 8, 2008, spanning the local influenza season, 796 subjects were enrolled. Six subjects withdrew before specimen collection; one was excluded due to specimen mislabeling. The remaining 789 subjects were analyzed; 199 provided a throat specimen, 145 provided a nasal specimen, and 445 provided both.

**Figure 1:**
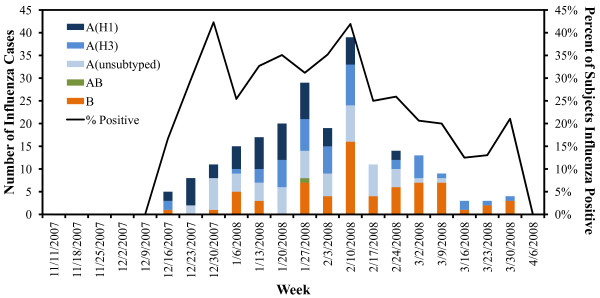
**Distribution of laboratory-confirmed influenza cases and overall influenza rate, during the 2007–2008 influenza season.** NOTE. Histogram depicts all laboratory-confirmed influenza cases in the study population, by type and subtype, throughout the duration of the study, along with the percentage of subjects who were positive for any influenza type by week.

Subject characteristics and outcomes are reported in Table 1. The overall influenza prevalence was 27.9%. Cases included 19.3% influenza A (44.0% A(H1), 39.7% A(H3), 16.4% unsubtypeable), 8.5% influenza B, and 1 influenza A/B co-infection. Of the 19 subjects with initially unsubtypeable influenza A infections, five subjects had sufficient remaining sample for further testing; all five subjects were positive for influenza A(H3) by PCR/ESI-MS (results not included in subsequent analyses). Distribution of cases throughout the season is illustrated in the Figure 1.

During the 2007–2008 season, laboratory-confirmed influenza rates were highest (33.0%) among 6- to 17-year-olds and lowest (18.5%) among 3- to 5-year-olds, though these differences were not significant (*p* = .110 across all age groups). The proportion of influenza A(H3) to A(H1) cases did not significantly differ across age groups (*p* = .623), but the proportion of influenza A to B differed (*p* = .019). No subjects reporting symptom duration ≥30 days (*n* = 14) had laboratory-confirmed influenza; 14 of 66 (21.2%) subjects reporting symptom duration of 14–29 days had influenza. Two subjects (0.3%), one influenza positive, denied a subjective fever and were eligible based solely on their recorded temperatures >38.0 °C; 577 subjects (73.1%) had both a cough and sore throat.

Laboratory-confirmed influenza rates varied from 21.2% at outpatient clinic 2 to 38.2% in the emergency room (*p* = .043 in multivariate model). Subjects contributing only one nasal specimen had a significantly lower rate of laboratory-confirmed influenza (17.9%) compared with those contributing one throat specimen only (29.7%) and one of each (30.3%) (*p* = .011 in multivariate model).

Bivariate regression revealed significant associations between laboratory-confirmed influenza and acute onset, fever, cough, body aches, and unvaccinated status in the whole population, 6- to 17-year-olds and 18- to 49-year-olds. These latter two age groups were subsequently combined due to these similarities (Table 2). Among 50- to 80-year-olds, only fever was predictive; in 3- to 5-year-olds, only unvaccinated status was predictive.

**Table 2:** Bivariate regression analysis of laboratory-confirmed influenza versus seven demographic and clinical variables of interest

Variable	Laboratory-confirmed influenza diagnosis							
	**Total study population**		**Subjects ages 3–5 years**		**Subjects ages 6–49 years**		**Subjects ages 50–80 years**	
	**(*n* = 789)**		**(*n* = 65)**		**(*n* = 523)**		**(*n* = 201)**	
	***n/*total (%)^a^**	**OR (95% CI)**	***n/*total (%)^a^**	**OR (95% CI)**	***n/*total (%)^a^**	**OR (95% CI)**	***n/*total (%)^a^**	**OR (95% CI)**
Continuous variable								
Age (years)		1.00 (0.99–1.01)		0.47 (0.17–1.10)		0.99 (0.98–1.01)		1.01 (0.95–1.07)
Categorical variables								
Sex								
Female	141/494 (28.5)	1.09 (0.78–1.53)	5/30 (16.7)	0.80 (0.18–3.38)	109/357 (30.5)	1.08 (0.71–1.66)	27/107 (25.2)	0.98 (0.50–1.96)
Male^b^	79/295 (26.8)	1.0	7/35 (20.0)	1.0	48/166 (28.9)	1.0	24/94 (25.5)	1.0
Acute onset								
≤3 days	110/273 (40.3)	2.49 (1.78–3.48)	8/30 (26.7)	2.77 (0.65–14.21)	86/199 (43.2)	2.71 (1.81–4.06)	16/44 (36.4)	1.98 (0.90–4.31)
>3 days^b^	110/516 (21.3)	1.0	4/35 (11.4)	1.0	71/324 (21.9)	1.0	35/157 (22.3)	1.0
Fever								
> 38.0°C	65/126 (51.6)	3.49 (2.31–5.27)	1/11 (9.1)	0.40 (0.01–3.37)	51/91 (56.0)	3.91 (2.39–6.44)	13/24 (54.2)	4.28 (1.63–11.51)
≤ 38.0°C^b^	155/663 (23.4)	1.0	11/54 (20.4)	1.0	106/432 (24.5)	1.0	38/177 (21.5)	1.0
Cough								
Yes	219/736 (29.8)	22.99 (3.72–890.52)	12/63 (19.0)	0.55 (≥0.04) ^c^	156/475 (32.8)	22.92 (3.84–932.74)	51/198 (25.8)	1.32 (≥0.14) ^c^
No^b^	1/53 (1.9)	1.0	0/2 (0.0)	1.0	1/48 (2.1)	1.0	0/3 (0.0)	1.0
Body aches^d^								
Yes	177/581 (30.5)	1.67 (1.13–2.50)	8/27 (29.6)	3.40 (0.79–17.59)	127/388 (32.7)	1.70 (1.06–2.79)	42/166 (25.3)	0.98 (0.40–2.57)
No^b^	43/207 (20.8)	1.0	4/37 (10.8)	1.0	30/135 (22.2)	1.0	9/35 (25.7)	1.0
Vaccinated								
Yes	39/217 (18.0)	0.47 (0.31–0.71)	0/17 (0.0)	0.13 (≤0.89) ^c^	16/112 (14.3)	0.32 (0.17–0.57)	23/88 (26.1)	1.07 (0.54–2.13)
No^b^	181/572 (31.6)	1.0	12/48 (25.0)	1.0	141/411 (34.3)	1.0	28/113 (24.8)	1.0

NOTE. Bivariate exact conditional logistic regression was performed in each age group. Bivariate results (OR, odds ratio; CI, confidence interval) are displayed here for all variables of interest (those that were retained in the final multivariate regression model in any age group) modeled against laboratory-confirmed influenza. ^a^Number of subjects meeting criteria at left who have laboratory-confirmed influenza/all subjects meeting criteria at left (percent). ^b^Reference category in regression model. ^c^OR is a median unbiased estimate; one limit of CI is infinite or crosses zero due to cell size of zero. ^d^Data missing for 1 subject age 3–5 years.

Multiple logistic regression in 6- to 49-year-olds showed the same pattern of associations with laboratory-confirmed influenza as bivariate analyses (Table 3). Neither enrollment site nor number and type of specimens tested altered the association between clinical predictors of interest and laboratory-confirmed influenza.

**Table 3:** Multivariate regression of laboratory-confirmed influenza versus demographic and clinical variables for subjects 6–49 years

**Variable**	**β estimate**	**OR (95% CI)**	***p* value**
Intercept term	−5.85		<.001
Age (years)	−0.00	1.00 (0.98–1.02)	.713
Sex	0.13		.606
Female		1.14 (0.70–1.84)	
Male^a^		1.0	
Acute onset	1.18		<.001
≤3 days		3.26 (2.09–5.10)	
>3 days^a^		1.0	
Fever	1.35		<.001
>38.0°C		3.84 (2.23–6.61)	
≤38.0°C^a^		1.0	
Cough	3.87		<.001
Yes		47.99 (6.29–366.13)	
No^a^		1.0	
Body aches	0.95		.001
Yes		2.59 (1.47–4.54)	
No^a^		1.0	
Vaccinated	−1.10		.001
Yes		0.33 (0.18–0.62)	
No^a^		1.0	

NOTE. CI, confidence interval; OR, odds ratio. Multivariate stepwise logistic regression was performed in the combined age group (*n* = 521). The reduced model is presented here (*n* = 523). Age and sex were retained in the final model despite not being statistically associated with the outcome. ^a^Reference category in regression model.

Table 4 displays the performance of 15 clinical case definitions as diagnostic tests constructed from combinations of acute onset, fever, cough, and body aches in 6- to 49-year-olds. Vaccination was not included given its variable clinical significance over influenza seasons. The highest specificity (98.6%) was noted with the all-4-criteria test; however, the sensitivity was 14.6%. The at-least-2-criteria test had a sensitivity of 94.9%, but a specificity of 20.2% and PPV of 33.8%. The CDC ILI case definition was most sensitive (50.8%, not shown) with the highest PPV (57.9%) among 6- to 17-year-olds and least sensitive among 3- to 5-year-olds (25.0%).

**Table 4:** **Performance metrics of influenza clinical case definitions in subjects 6–49 years (*n*** **= 523)**

		**Laboratory influenza**					
	**Clinical test**	**diagnosis**					
**Clinical test**	**result**	**Positive**	**Negative**	**SN (%) (95% CI)**	**SP (%) (95% CI)**	**PPV (%) (95% CI)**	**NPV (%) (95% CI)**
At least 1 criterion^a^	Positive	157	363	100.0 (undefined) ^b^	0.8 (0.0–1.6)	30.2 (26.3–34.1)	100.0 (undefined) ^b^
	Negative	0	3				
At least 2 criteria^a^	Positive	149	292	94.9 (93.0–96.8)	20.2 (16.8–23.7)	33.8 (29.7–37.8)	90.2 (87.7–92.8)
	Negative	8	74				
At least 3 criteria^a^	Positive	91	73	58.0 (53.7–62.2)	80.1 (76.6–83.5)	55.5 (51.2–59.7)	81.6 (78.3–84.9)
	Negative	66	293				
All 4 criteria^a^	Positive	23	5	14.6 (11.6–17.7)	98.6 (97.6–99.6)	82.1 (78.9–85.4)	72.9 (69.1–76.7)
	Negative	134	361				
*Exact 2 criteria*^*c*^							
Acute onset and fever	Positive	31	22	19.7 (16.3–23.2)	94.0 (92.0–96.0)	58.5 (54.3–62.7)	73.2 (69.4–77.0)
	Negative	126	344				
Acute onset and cough	Positive	86	85	54.8 (50.5–59.0)	76.8 (73.2–80.4)	50.3 (46.0–54.6)	79.8 (76.4–83.3)
	Negative	71	281				
Acute onset and body aches	Positive	64	68	40.8 (36.6–45.0)	81.4 (78.1–84.8)	48.5 (44.2–52.8)	76.2 (72.6–79.9)
	Negative	93	298				
Fever and cough	Positive	51	29	32.5 (28.5–36.5)	92.1 (89.8–94.4)	63.8 (59.6–67.9)	76.1 (72.4–79.7)
	Negative	106	337				
Fever and body aches	Positive	42	25	26.8 (23.0–30.5)	93.2 (91.0–95.3)	62.7 (58.5–66.8)	74.8 (71.1–78.5)
	Negative	115	341				
Cough and body aches	Positive	126	224	80.3 (76.8–83.7)	38.8 (34.6–43.0)	36.0 (31.9–40.1)	82.1 (78.8–85.4)
	Negative	31	142				
Cough + any of other 3 criteria	Positive	149	264	94.9 (93.0–96.8)	27.9 (24.0–31.7)	36.1 (32.0–40.2)	92.7 (90.5–95.0)
	Negative	8	102				
*Exact 3 criteria*^*c*^							
Acute onset, fever, cough	Positive	31	14	19.7 (16.3–23.2)	96.2 (94.5–97.8)	68.9 (64.9–72.9)	73.6 (69.9–77.4)
	Negative	126	352				
Acute onset, cough, body aches	Positive	64	47	40.8 (36.6–45.0)	87.2 (84.3–90.0)	57.7 (53.4–61.9)	77.4 (73.8–81.0)
	Negative	93	319				
Acute onset, fever, body aches	Positive	23	9	14.6 (11.6–17.7)	97.5 (96.2–98.9)	71.9 (68.0–75.7)	72.7 (68.9–76.5)
	Negative	134	357				
Fever, cough, body aches	Positive	42	18	26.8 (23.0–30.5)	95.1 (93.2–96.9)	70.0 (66.1–74.0)	75.2 (71.5–78.9)
	Negative	115	348				
*CDC ILI case definition*^*d*^							
Total study population	Positive	83	82	37.7 (33.6–41.9)	85.6 (82.6–88.6)	50.3 (46.0–54.6)	78.0 (74.5–81.6)
	Negative	137	487				
Subjects ages 3–5 years	Positive	3	15	25.0 (21.3–28.7)	71.7 (67.8–75.6)	16.7 (13.4–19.9)	80.9 (77.5–84.2)
	Negative	9	38				
Subjects ages 6–49 years	Positive	63	52	40.1 (35.9–44.3)	85.8 (82.8–88.8)	54.8 (50.5–59.0)	77.0 (73.4–80.6)
	Negative	94	314				
Subjects ages 50–80 years	Positive	17	15	33.3 (29.3–37.4)	90.0 (87.4–92.6)	53.1 (48.8–57.4)	79.9 (76.4–83.3)
	Negative	34	135				

NOTE. CDC, Centers for Disease Control and Prevention; CI, confidence interval; ILI, influenza-like illness; NPV, negative predictive value; PPV, positive predictive value; SN, sensitivity; SP, specificity. The first 15 clinical tests displayed are defined according to combinations of 4 influenza predictors (“criteria”) present in a subject: acute onset, fever, cough, and body aches. CI calculated for a proportion estimate as follows: (estimate) ± 1.96 x √(estimate x (1 – estimate)/n). ^a^Positive test defined by at least *n* of the 4 criteria of interest being met. ^b^CI undefined; cannot be calculated for a proportion of 1.0. ^c^The exact criteria listed are met, regardless of whether the other unlisted criteria are met. ^d^CDC ILI definition = temperature ≥37.8 °C and either cough or sore throat in absence of another known cause. Presence of another known cause is not assessable in this study. This case definition’s performance was evaluated in and is reported for all age groups included in the study.

Bivariate logistic regression modeling of influenza B versus A in 6- to 49-year-olds (*n =* 156) revealed significant or borderline associations with age, smoking, acute onset, fever, and vaccination. In multiple logistic regression modeling positive smoking history was associated with increased odds of influenza A versus B (adjusted odds ratio [OR], 8.95; 95% CI, 1.99–40.24). Age in 1-year units (OR, 1.07; 95% CI, 1.03–1.11) and vaccinated status (OR, 4.51; 95% CI, 1.19–17.09) were associated with influenza B versus A. Bivariate modeling of influenza A(H3) versus A(H1) in the same age group (*n =* 75) demonstrated no significant differences. However, the multivariate model demonstrated an association between positive smoking history and influenza A(H3) (OR, 10.47; 95% CI, 1.28–85.46) and fever associated with influenza A(H1) (OR, 4.48; 95% CI, 1.11–18.12).

## Discussion

This study compared the predictive values of demographic and clinical factors for laboratory-confirmed influenza during the 2007–2008 influenza season in Southern California among different age groups and evaluated the performance of resulting case definitions. Marked variations across age groups were found with robust predictors among 6- to 49-year-olds and few predictors among younger children and older adults. No single case definition was found to have both a sensitivity and specificity of clinical utility, even in an age group exhibiting the strongest predictors; the CDC ILI case definition would have excluded 62% of influenza cases in this study population. With no evidence for a diagnostically useful case definition (used alone) and the potential unreliability of RIDTs [12,36], new diagnostic strategies are urgently needed.

In our study population, 6- to 49-year-olds had the highest influenza rate (differences not statistically significant), with strong associations found between laboratory-confirmed influenza and acute onset, fever, cough, body aches, and unvaccinated status, consistent with prior studies [16,17,21,23,25,37]. Cough had the strongest association with influenza. Influenza positive 6- to 49-year-olds had approximately three times the odds of being unvaccinated (*n* = 141, 89.8%) compared to negative subjects (*n* = 270, 73.8%) (*p* = .001), suggesting a moderate protective effect of the vaccine for this season in this age group. No demographic or clinical predictors of influenza were found among 3- to 5-year-olds, consistent with findings in similar studies [27]. The difficulty of eliciting a symptomatic history when the subject has limited language skills may contribute to this phenomenon [38]. Vaccination appeared strongly protective in this age group. A (non-significant) lower rate of influenza in this group conflicts with the common belief that young children exhibit a higher incidence of influenza, higher viral loads and higher persistence of the virus. However, CDC data from outpatients with ILI in 12 states during the 2010–2011 influenza season suggests that the rate of laboratory-confirmed influenza was lower among children < 5 years old compared to 5- to 17-year-olds [39]. Among 50- to 80-year-olds, only fever was associated with influenza, consistent with prior studies failing to identify reliable clinical predictors among older adults [20,24]. No protective effect from vaccination was apparent in this group, as might be expected with age-related waning immunity [40], although denominators were small.

Together, these findings substantiate previous observations that during a community influenza epidemic, acute onset of fever and cough in young to middle-aged adults are the best individual clinical predictors for laboratory-confirmed influenza [17,21], with young children demonstrating the fewest predictors [27]. Differing influenza detection rates according to specimen type collected warrants further study.

The all-4-criteria clinical case definition (requiring acute onset, fever, cough, and body aches) had a strong specificity among 6- to 49-year-olds (98.6%), but missed 85% of influenza cases. The at-least-2-criteria test (subject must have at least two of the four criteria) captured 94.9% of cases, but only excluded influenza in 20.2% of noncases; 33.8% of test positives had influenza in this group (overall prevalence similar to that of a typical influenza epidemic). The clinical tests defined by exactly two or three criteria likewise performed poorly, though roughly half had higher sensitivity than the CDC ILI case definition (40.1%) in this age group. The CDC ILI definition’s sensitivity varied significantly across age groups, with the poorest performance among 3- to 5-year-olds and the highest among 6- to 49-year-olds. Across the whole population the CDC ILI definition missed >60% of cases and had a PPV <60% in each age group. NPVs ranged from 73-100%, indicating that the absence of most of the symptoms modeled effectively predicted the absence of influenza infection; the at-least-1-criterion test, at-least-2-criteria test, and “cough + any of other 3 criteria” test could all be utilized to exclude influenza (NPV >90%), eliminating the need for a follow-up rapid or laboratory-based test.

All case definitions in Table 4 performed inferiorly to the typically cited sensitivities (50%–70%) or specificities (90%–95%) or both of RIDTs [36]. However, a common RIDT was found to have a sensitivity as low as of 27% (95% CI, 19%–32%) for influenza during 2007–2008 [12]; recent studies of that same and other RIDTs report sensitivities 38%–69% in detection of pH1N1 [13-15] and 31%–63% against other strains [14]. If RIDTs are less sensitive than previously cited, their optimal diagnostic utility might be among groups with higher influenza prevalence (eg, patients meeting a sensitive clinical case definition like the at-least-2-criteria clinical test during a local epidemic) with confirmatory testing following negative RIDT results [36].

Vaccination status was excluded in this diagnostic modeling since the effect of vaccination is likely to change annually with variable strain matching. Clinicians should take vaccination status into account when making a clinical diagnosis only once the performance of the vaccine has been established for that region and season.

Influenza type/subtype analysis yielded unexpected results. The meaning of the associations of influenza A with history of smoking and influenza B with increasing age is not clear. Researchers previously reported the two influenza types might infect different age groups at different rates, but may cause a similar clinical syndrome across age groups [22]. Differences in rates across age groups may be related to previous circulation of similar strains. Smoking increases with age among 6- to 49-year-olds; thus confounding is unlikely. Fever was associated with influenza A(H1) infection compared with A(H3). If fever correlates with a higher viral titer, it is not surprising that the initially unsubtypeable specimens were A(H3)-positive. However, if fever reflects more severe or systemic disease, this finding contradicts previous reports suggesting influenza A(H3) was associated with such illness [16,41]. We are unaware of a physiologic mechanism through which smoking would increase the risk of influenza A(H3), or decrease the risk of A(H1), which our results suggest, but this merits further investigation.

This study’s major strengths include a methodology facilitating comparison to similar studies and inclusion in meta-analyses and an analysis of age group and influenza type/subtype effects. The surveillance population studied provides an ideal setting for this analysis: a large outpatient population at the point of care, wide age range and socioeconomic spectrum, broad and uniform inclusion criteria (resulting in symptomatic diversity), and robust laboratory influenza diagnosis with type and subtype. Type distributions were similar to national (71% influenza A and 29% influenza B) [35] and local [42] rates in the same season. The military dependent population studied is similar to the general US population, though dependents may be healthier due to universal access to medical care.

Study limitations include omission of subjects ages <3 years and incomplete data on prior vaccination history in children—potential receipt of two doses in individuals ages ≤8 years was not documented. Other limitations include use of self-reported vaccination data, lack of antiviral or antipyretic use history, lack of information regarding comorbid conditions that increase risk of influenza or adverse outcomes from influenza, and asymmetry between influenza A and B testing methods. Subjects in this study may not be considered high risk for influenza complications, thus may not represent the population for whom antiviral therapy is clearly indicated for treatment of influenza infection. Clinical predictors of influenza and the performance of clinical case definitions may differ in a high risk population. An RT-PCR assay for influenza B was not utilized, thus true influenza B and overall influenza rates may be underestimated. We believe underdiagnosis of influenza B is more likely to create a type II error. As the clinical case definitions modeled here were created utilizing our entire data set, sensitivities and specificities could be overestimates of the true values; these models should be evaluated and validated in other populations and during other time periods. Last, we report on only one influenza season and cannot assess clinical disease variations over time resulting from antigenic drift.

## Conclusions

This study demonstrates that clinical parameters strongly predict influenza in a diverse local population and are age-dependent; but clinical case definitions used alone, including the CDC ILI case definition, may not sufficiently distinguish influenza from non-influenza during an epidemic. Case definitions could still augment diagnosis and management for 6- to 49-year-old patients during an influenza season: (1) screening with a sensitive case definition followed by RIDT or definitive testing when positive or (2) screening otherwise healthy outpatients with a specific case definition. These strategies could enhance appropriate antimicrobial use. As always, the effect of varying seasonal and geographic rates on diagnostic predictive values should be considered. The 2009 emergence of pH1N1 highlights the urgent need for reevaluation of RIDTs and further investigation into the changing clinical disease spectrum of seasonal and novel influenza strains. An evaluation of clinical case definitions in high-risk groups, more likely to benefit from antiviral therapy, is needed. Standardization of clinical case definitions and clinical sampling techniques would facilitate better comparisons between studies and more effective evaluation of influenza diagnostics.

## Competing interests

The authors declare that they have no competing interests.

## Authors’ contributions

TW participated in the design of the study, coordinated study activities, supervised enrollment and data collection, conducted data analysis, and drafted the manuscript. DF conceived of the study and participated in its design, supervised laboratory reference testing, and participated in manuscript revision. SB participated in the design of the study and participated in manuscript revision. HL participated in the design of the study and assisted with data analysis. PB and JW participated in manuscript revision. All authors read and approved the final manuscript. TW is a senior medical epidemiologist at the Naval Health Research Center (NHRC). SB is a researcher at NHRC and a professor and Epidemiology Division Head at San Diego State University (SDSU) Graduate School of Public Health (GSPH). HL is an assistant professor at SDSU GSPH. JW is a faculty member of the UCSD/SDSU Joint General Preventive Medicine Residency Program. PB is department head for the NHRC Department of Operational Infectious Diseases (OID). DF was assistant department head for NHRC OID at the time of manuscript submission. All authors read and approved the final manuscript.

## Acknowledgments

We are grateful to research associates Meghan McClure, Chasity Greer, and Korinna David, and laboratory staff at the Naval Health Research Center who performed all subject enrollments and testing, without whose support such studies could not be conducted. Thanks also to the Naval Medical Center San Diego for supporting and facilitating the establishment of an ongoing influenza surveillance network in its health care system and to the staff in subordinate clinics for their cooperation with this work.

This work represents reports 11–16 supported by the Armed Forces Health Surveillance Center, Global Emerging Infections Surveillance and Response System (GEIS) operations [work unit number 60501]. The funding body was not involved in study design, data collection, analysis, interpretation of results or manuscript writing.

## Pre-publication history

The pre-publication history for this paper can be accessed here:

http://www.biomedcentral.com/1471-2334/12/129/prepub
